# Impact of HBcrAg levels on HBsAg seroconversion after HBV rebound: a case report

**DOI:** 10.1186/s40780-023-00321-x

**Published:** 2023-12-19

**Authors:** Maki Ohkubo, Kuniaki Fukuda, Shigeru Chiba, Masato Homma

**Affiliations:** 1 Department of Pharmacy, University of Tsukuba Hospital, Ibaraki, Japan; 2 Department of Pharmaceutical Sciences, Graduate School of Comprehensive Human Sciences, University of Tsukuba, Ibaraki, Japan; 3 Department of Gastroenterology, Faculty of Medicine, University of Tsukuba, Ibaraki, Japan; 4 Department of Hematology, Faculty of Medicine, University of Tsukuba, Ibaraki, Japan

**Keywords:** HBsAg seroconversion, HBcrAg levels, HBV reactivation, HBV rebound

## Abstract

**Background:**

Nucleoside analogues (NAs) such as entecavir are required for at least 12 months when patients with resolved hepatitis B virus (HBV) infection develop HBV reactivation. Entecavir treatment does not always achieve hepatitis B surface antigen (HBsAg) seroconversion. The cessation of NA for HBV reactivation sometimes causes HBV rebound. The impact of hepatitis B core-related antigen (HBcrAg) on predicting HBV rebound is controversial.

**Case presentation:**

A 67-year-old woman with resolved HBV infection received rituximab for post-transplant lymphoproliferative disorder after peripheral blood stem cell transplantation. Since she tested positive for HBV-DNA after the first rituximab therapy (day 0), entecavir treatment was started. Because the HBV-DNA test became negative and her liver function had been normal, entecavir was terminated on day 376. According to the retrospective measurements, HBcrAg remained positive while the HBV-DNA level was undetectable. One hundred forty-one days after entecavir cessation, the HBV-DNA turned positive again, suggesting HBV rebound (day 517). Her liver function deteriorated and HBV infection worsened, even though entecavir treatment was resumed on day 615. On the contrary, hepatitis B surface antibody levels increased after the rebound, resulting in HBsAg seroconversion with HBcrAg and HBV-DNA levels undetectable. HBV reactivation has not been detected after the second entecavir cessation, and both HBcrAg and HBV-DNA levels remained undetectable.

**Discussion and conclusions:**

This case suggests that NA cessation induced-HBV rebound achieved HBsAg seroconversion under the guidance of a hepatologist. Since HBcrAg had been detectable while HBV-DNA was undetectable, HBcrAg may be an index for predicting HBV rebound resulting in HBsAg seroconversion as well as other conventional laboratory tests. Prospective measuring HBcrAg is required to confirm this case report.

## Background

Hepatitis B virus (HBV) infection is still endemic in Asia-Pacific countries, affecting 2–4% of the population aged 19–49 years [1]. HBV reactivation, caused by immunosuppressive therapies in patients with HBV infection, is associated with the risk of severe hepatic dysfunction [2], resulting in death in some cases. The guideline recommends monitoring HBV-DNA levels in patients with resolved HBV during and after chemotherapy [2]. Nucleoside analogues (NAs) such as entecavir are required for at least 12 months when patients with resolved HBV infection develop HBV reactivation, occasionally resulting from intensive immunosuppressive treatment including rituximab [2]. However, entecavir treatment does not always achieve hepatitis B surface antigen (HBsAg) seroconversion [3].

Herein, we report a patient who underwent peripheral blood stem cell transplantation (PBSCT) and showed HBsAg seroconversion after a rebound of HBV infection due to the cessation of entecavir for HBV reactivation. After the patient achieved HBsAg seroconversion, we confirmed a change in her hepatitis B core-related antigen (HBcrAg), retrospectively, which consisted of three types of antigen structural proteins, namely, hepatitis B core antigen, hepatitis B e antigen (HBeAg) and p22cr antigen [2]. HBcrAg may be a suitable marker for predicting HBV rebound as well as other conventional laboratory tests. This paper will discuss the impact of HBcrAg monitoring on predicting a rebound of HBV infection.

## Case presentation

**Fig. 1:**
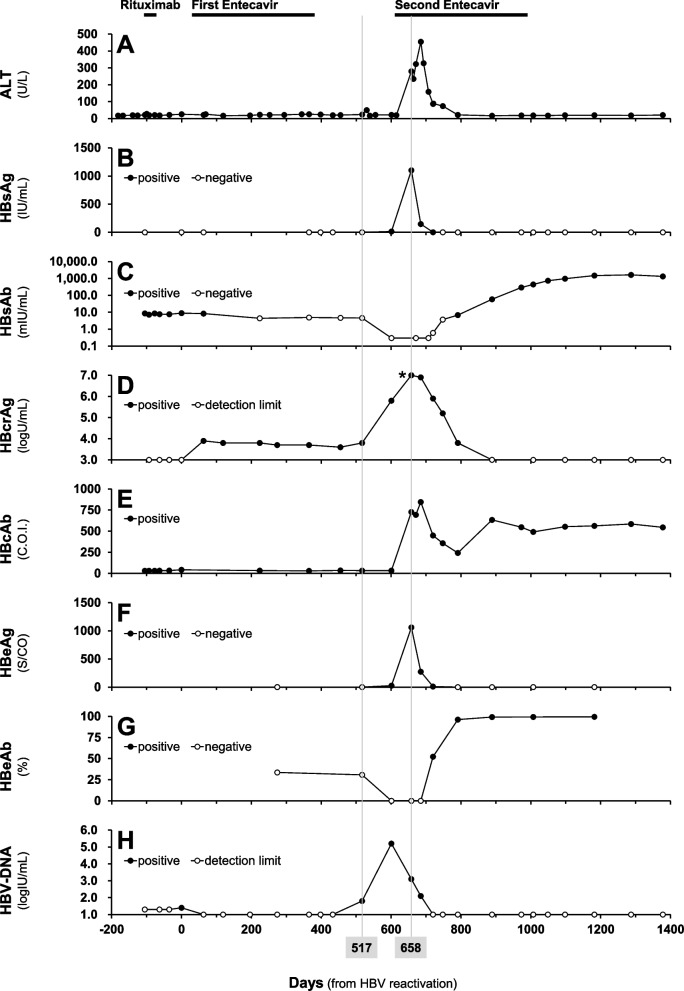
Changes in biochemical and virological parameters in the course of HBsAg seroconversion. The detection limits of HBV-DNA (**H**) before and after the HBV reactivation were 1.3 and 1.0 LogIU/mL, respectively. *: HBcrAg level was 7.0 LogU/mL (above the detection upper limit). ALT (**A**), alanine aminotransferase; HBsAg (**B**), hepatitis B surface antigen; HBsAb (**C**), hepatitis B surface antibody; HBcrAg (**D**), hepatitis B core-related antigen; HBcAb (**E**), hepatitis B core antibody; HBeAg (**F**), hepatitis B e antigen; HBeAb (**G**), hepatitis B e antibody

A 67-year-old woman underwent PBSCT for therapy-related acute myeloid leukaemia and received azacitidine, busulfan and fludarabine therapy. Before PBSCT, she had been diagnosed with resolved HBV infection; HBsAg, negative; hepatitis B core antibody (HBcAb), positive (180.6 C.O.I.); and hepatitis B surface antibody (HBsAb), positive (36.9 mIU/mL). She had no history of HBV vaccination, but she experienced acute hepatitis B caused by blood transfusion for her child birth before PBSCT. Changes in HBsAb and HBcrAg during the course are presented in Fig. 1, as well as other liver function and viral status, alanine aminotransferase (ALT), HBsAg, HBcAb, HBeAg, hepatitis B e antibody (HBeAb) and HBV-DNA levels. Serum HBcrAg levels were determined via chemiluminescent enzyme immunoassay (LUMIPULSE®, Fujirebio Inc., Tokyo, Japan).

She received rituximab for post-transplant lymphoproliferative disorder 26 months after PBSCT, her HBsAb decreased (8.6 mIU/mL) and HBV-DNA increased slightly to detectable though the levels of < 1.3 logIU/mL. Since she tested positive for HBV-DNA (1.4 logIU/mL) 103 days after the first rituximab therapy (day 0, Fig. 1H), the scheduled rituximab administration was skipped and the first entecavir treatment was started according to the Japanese guideline for HBV reactivation [2]. Immediately after the first entecavir treatment, the HBV-DNA test became negative (day 63, Fig. 1H). Since her liver function (ALT levels) had been normal (Fig. 1A) and both HBsAg and HBV-DNA remained negative during NA treatment (Fig. 1B and H), entecavir was terminated on day 376. According to the retrospective measurements, HBcrAg remained positive (3.6–3.9 logU/mL) while the HBV-DNA level was undetectable under the first entecavir treatment (day 63–455, Fig. 1D). One hundred forty-one days after entecavir cessation, the HBV-DNA test turned positive again (1.8 logIU/mL), suggesting HBV rebound (day 517). Her HBV-DNA level reached 5.2 logIU/mL (day 601, Fig. 1H), her liver function deteriorated and HBV infection worsened; ALT, HBsAg, HBcrAg, HBcAb and HBeAg were elevated to high levels at 280 U/L, 1101.8 IU/mL, 7.0 logU/mL (above the detection range), 727.3 C.O.I. and 1060 S/CO, respectively (day 658, Fig. 1A, B, D, E and F), even though entecavir treatment was resumed on day 615. The HBsAb level, which had been negative before the HBV rebound, further decreased (0.3 mIU/mL) when the HBV rebound was detected (Fig. 1C). ALT peaked at 455 U/L on day 685 when the levels for HBsAg, HBcrAg, HBeAg and HBV-DNA peaked out and declined (Fig. 1A, B, D, F and H). On the contrary, HBsAb and HBeAb levels increased to 292.8–1631.6 mIU/mL and 99.2–99.5%, respectively (Fig. 1C and G), after the rebound, resulting in HBsAg seroconversion with HBcrAg and HBV-DNA levels undetectable. The second entecavir treatment was terminated on day 986. HBV reactivation has not been detected 392 days after the second entecavir cessation, and both HBcrAg and HBV-DNA levels remained undetectable. No difference in medication adherence and renal function was observed between first and second entecavir treatment. Daily dose of entecavir was 0.5 mg for both first and second treatment.

## Discussion and conclusions

This case suggests that NA cessation induced-HBV rebound achieved HBsAg seroconversion under the guidance of a hepatologist, and that among the conventional laboratory data, HBcrAg may be a useful index for HBV rebound after NA cessation. The risk of severe hepatitis should be assessed carefully by using HBsAg levels and liver function when HBV rebound.

A study reported that HBsAg seroconversion was achieved by corticosteroid withdrawal-induced rebound followed by interferon administration, although the rebound was life-threatening [4]. Because current NA such as entecavir has a potent anti-HBV activity compared with interferon, rebound for HBsAg seroconversion can be successfully achieved by NA intervention, as in the present case. We presume that her liver injury like the acute hepatitis B due to cessation of first entecavir, eventually changed HBV immune status to the pattern of post-acute hepatitis B (rebound).

HBcrAg positivity (3.6–3.9 logU/mL) while HBV-DNA is undetectable may be a marker for determining whether a rebound is indicated. In patients with chronic HBV infection undergoing lamivudine treatment, HBcrAg levels decline slowly compared with HBV-DNA levels [5]. False-negative HBV-DNA under entecavir therapy may be observed even though HBV are surviving because intracellular entecavir 3-phosphate inhibits reverse transcriptase from transcribing RNA to DNA in determining serum HBV-DNA [2]. On the other hand, determining HBcrAg is not affected by the activity of reverse transcriptase in the entecavir user [2]. It is considered that HBcrAg positivity indicates HBV remain alive in the liver even though serum HBV-DNA levels are undetectable.

The currently developed high-sensitivity HBcrAg assay (lower detection limit of 2.1 logU/mL) detected HBcrAg in 9 of 13 patients with HBV reactivation before their HBV-DNA test turned positive [6]. Interestingly, this assay can identify the patients earlier, who would develop HBV rebound after NA cessation [6]. Therefore, the monitoring HBcrAg and HBV-DNA levels is recommended to assess the virus status in patients receiving entecavir for preventing HBV reactivation. A limitation of the present case study is measuring HBcrAg retrospectively for evaluating the HBV rebound. Prospective measuring HBcrAg will be required to confirm our hypothesis in future study.

## Data Availability

All data generated or analysed during this study are included in this published article.

## Abbreviations

HBV: Hepatitis B virus
NA: Nucleoside analogue
PBSCT: Peripheral blood stem cell transplantation
ALT: Alanine aminotransferase
HBsAg: Hepatitis B surface antigen
HBsAb: Hepatitis B surface antibody
HBcrAg: Hepatitis B core-related antigen
HBcAb: Hepatitis B core antibody
HBeAg: Hepatitis B e antigen
HBeAb: Hepatitis B e antibody
